# Supplementary material for: Variation in Glucose-6-Phosphate Dehydrogenase activity following acute malaria
Source: PLoS Negl Trop Dis. 2022 May 11;16(5):e0010406. doi: 10.1371/journal.pntd.0010406 (PMC9094517; doi:10.1371/journal.pntd.0010406)
Supplement: S1 Table — (DOCX) [file pntd.0010406.s001.docx]

| **Country** | **Protocol title** | **Local board** | **Approval number** | **Australian board** | **Approval number** | **Clinical Trials.gov. reference number** |
| --- | --- | --- | --- | --- | --- | --- |
| **Bangladesh** | A study to assess primaquine treatment guidelines for malaria in south-east Bangladesh | Ethical Review Committee of the ICDDR,B | 14053 | Human Research Ethics Committee of the Northern Territory | 14.2228 | NCT02389374 |
| **Bangladesh** | The effect of an acute malaria episode on G6PD activity (MALAGA) | Ethical Review Committee of the ICDDR,B | 16058 | Human Research Ethics Committee of the Northern Territory | 16.2615 | --- |
| **Indonesia** | A randomized controlled trial on malaria primaquine treatment in Timika, Indonesia (TRIPI) | The Medical and Health Research Ethics Committee | KE/FK/522/EC/2016 | Human Research Ethics Committee of the Northern Territory | 15.2517 | NCT02787070 |
| **Ethiopia** | Improving the radical cure of vivax malaria: A multicenter randomized, placebo -controlled comparison of short and long course primaquine regimens (IMPROV) and G6PD sub-study | The National Research Ethics Review Committee | --- | Human Research Ethics Committee of the Northern Territory | 13.1991 | NCT01814683 |
