# Supplementary material for: Variation in Glucose-6-Phosphate Dehydrogenase activity following acute malaria
Source: PLoS Negl Trop Dis. 2022 May 11;16(5):e0010406. doi: 10.1371/journal.pntd.0010406 (PMC9094517; doi:10.1371/journal.pntd.0010406)
Supplement: S2 Table — * This trial is complemented by an observational arm with a total sample size of 420 that is not registered with Clinicaltrials.gov. *** Not applicable for complementary arm **this site is part of a multi-center trial, however measurements relevant to this study were only performed at this site. (DOCX) [file pntd.0010406.s002.docx]

| **Country** | **Study Name** | **Clinicaltrials.gov Identifier** | **Study design** | **Study drugs** | **Inclusion criteria** | **Enrolment period** | **Total enrolled** | **Total included in this study (%)** |
| --- | --- | --- | --- | --- | --- | --- | --- | --- |
| **Bangladesh** | A Study to Assess Safety of Current Standard Malaria Treatment and an Assessment of G6PD Status in South-east Bangladesh | NCT02389374 | Observational | - Artemether-Lumefantrine - Single dose Primaquine (0.25mg/kg) - Chloroquine - Primaquine radical cure (3.5mg/kg for 14days) | - Consent - Age ≥ 12 months - Weight ≥ 5kg - *P. falciparum* and/or *P. vivax* infection - Fever (37.5°C) / history of fever in last 48 hours - Hb ≥ 8g/dL | Sep. 2014 to Feb. 2015 | 181 | 99 (54.7) |
| **Indonesia*** | A Randomized Controlled Trial on Malaria Primaquine Treatment in Timika, Indonesia (TRIPI) | NCT02787070 | Randomized controlled trial | - Dihydroartemisinin and Piperaquine - Primaquine radical cure (7mg/kg for 14 days, supervised or unsupervised) | - Consent - Age ≥12 months - Weight ≥ 5kg - *P. falciparum* and/or *P. vivax* infection - Hb≥9g/dL - G6PD normal by fluorescent spot test (only for clinical trial arm)* | Oct. 2016 to May. 2018 | 664* | 75 (11.3%) |
| **Ethiopia***** | IMPROV - Ethiopia | NCT01814683 | Randomized controlled trial | - Chloroquine - Primaquine (7mg/kg for 7 or 14 days) | - Consent - Age ≥ 6 months - Weight ≥ 5kg - *P. vivax* infection - Fever (37.5°C) / history of fever in last 48 hours - G6PD normal by fluorescent spot test | Jan 2017 to Oct. 2017 | 374 | 173 (46.3%) |
