# Supplementary material for: Variation in Glucose-6-Phosphate Dehydrogenase activity following acute malaria
Source: PLoS Negl Trop Dis. 2022 May 11;16(5):e0010406. doi: 10.1371/journal.pntd.0010406 (PMC9094517; doi:10.1371/journal.pntd.0010406)
Supplement: S3 Table — (DOCX) [file pntd.0010406.s003.docx]

|  | **Bangladesh** | **Indonesia** | **Ethiopia** |
| --- | --- | --- | --- |
| **1^st^ enrolment period** | 20.08.14 – 29.01.15 | 31.10.16 – 17.11.17 | 26.01.17 – 08.04.17 |
| **2^nd^ enrolment period** | 20.03.17 – 15.05.17 | 20.03.17 – 04.05.18 | 20.07.17 – 03.10.17 |
| **Inclusion criteria** | - G6PD measurement during clinical trial - Contactable - Willing to participate - Aparasitaemic by blood film or RDT | - G6PD measurement during clinical trial - Contactable - Willing to participate - Aparasitaemic by blood film or RDT | - G6PD measurement during clinical trial - Contactable - Willing to participate - Aparasitaemic by blood film or RDT |
| **Selection criteria** | Patients with lowest activities revisited | Patients enrolled in clinical trial | Patients enrolled in clinical trial |
| **Proportion of patients from first study revisited (in %)** | 48.1 | 11.3 | 46.3 |
| **Malaria diagnosis 1^st^ visit** | Microscopy | Microscopy | Microscopy |
| **Malaria diagnosis 2nd visit** | Carestart RDT (USA) | Microscopy | Microscopy |
| **Spectrophotometry assay** | Randox Laboratories (PD410, UK) | Trinity Biotech (345-A, Ireland) | Trinity Biotech (345-A, Ireland) |
| **Assay temperature (°C)** | 37°C | 30°C | 30°C |
| **Spectrophotometer** | Shimadzu 1800 (Shimadzu, Japan) | Shimadzu 1800 (Shimadzu, Japan) | Humalyzer 3000 (Human, Germany) |
| **Normal Controls** | Randox Laboratories (PD2618, UK) | Trinity Biotech (G6888, Ireland) | Trinity Biotech (G6888, Ireland) |
| **Intermediate Controls** | Not done | Trinity Biotech (G5029, Ireland) | Trinity Biotech (G5029, Ireland) |
| **Deficient Controls** | Randox Laboratories (PD2617, UK) | Trinity Biotech (G5888, Ireland) | Trinity Biotech (G5888, Ireland) |
| **Hemoglobin measurement** | Hemocue 301, (Hemocue, Sweden) | Hemocue 301, (Hemocue, Sweden) | Hemocue 301, (Hemocue, Sweden) |
