# Supplementary material for: Variation in Glucose-6-Phosphate Dehydrogenase activity following acute malaria
Source: PLoS Negl Trop Dis. 2022 May 11;16(5):e0010406. doi: 10.1371/journal.pntd.0010406 (PMC9094517; doi:10.1371/journal.pntd.0010406)
Supplement: S4 Table — (DOCX) [file pntd.0010406.s004.docx]

| **Country** | **Control** | **n** | **Mean (U/gHb)** | **95%CI** | **Coefficient of variation** |
| --- | --- | --- | --- | --- | --- |
| **Bangladesh** | Def. | 89 | 1.3 | 1.2 to 1.4 | 0.4 |
|  | Normal | 89 | 8.7 | 8.5 to 8.8 | 0.1 |
| **Indonesia** | Def. | 42 | 0.7 | 0.6 to 0.7 | 0.2 |
|  | Interm. | 42 | 3.4 | 3.3 to 3.5 | 0.1 |
|  | Normal | 42 | 10.3 | 10.0 to 10.5 | 0.1 |
| **Ethiopia** | Def. | 28 | 1.2 | 1.1 to 1.2 | <0.1 |
|  | Interm. | 28 | 4.9 | 4.8 to 4.9 | <0.1 |
|  | Normal | 28 | 12.8 | 12.6 to 13.0 | 0.1 |
