# Supplementary material for: Variation in Glucose-6-Phosphate Dehydrogenase activity following acute malaria
Source: PLoS Negl Trop Dis. 2022 May 11;16(5):e0010406. doi: 10.1371/journal.pntd.0010406 (PMC9094517; doi:10.1371/journal.pntd.0010406)
Supplement: S5 Table — (DOCX) [file pntd.0010406.s005.docx]

|  | **Number with <30% activity during follow up** | **Number with ≥30% to <70% during follow up** | **Number with ≥70% during follow up** | **Total** |
| --- | --- | --- | --- | --- |
|  | **Bangladesh** | | |  |
| Male | 6 | 21 | 35 | **62** |
| Female | 0 | 12 | 13 | **25** |
|  | **Ethiopia** | | |  |
| Male | 0 | 1 | 94 | **95** |
| Female | 0 | 0 | 78 | **78** |
|  | **Indonesia** | | |  |
| Male | 0 | 2 | 38 | **40** |
| Female | 0 | 3 | 32 | **35** |
| **Total** | **6** | **39** | **300** | **335** |
