# Supplementary figures and images for: Variation in Glucose-6-Phosphate Dehydrogenase activity following acute malaria
Source: PLoS Negl Trop Dis. 2022 May 11;16(5):e0010406. doi: 10.1371/journal.pntd.0010406 (PMC9094517; doi:10.1371/journal.pntd.0010406)

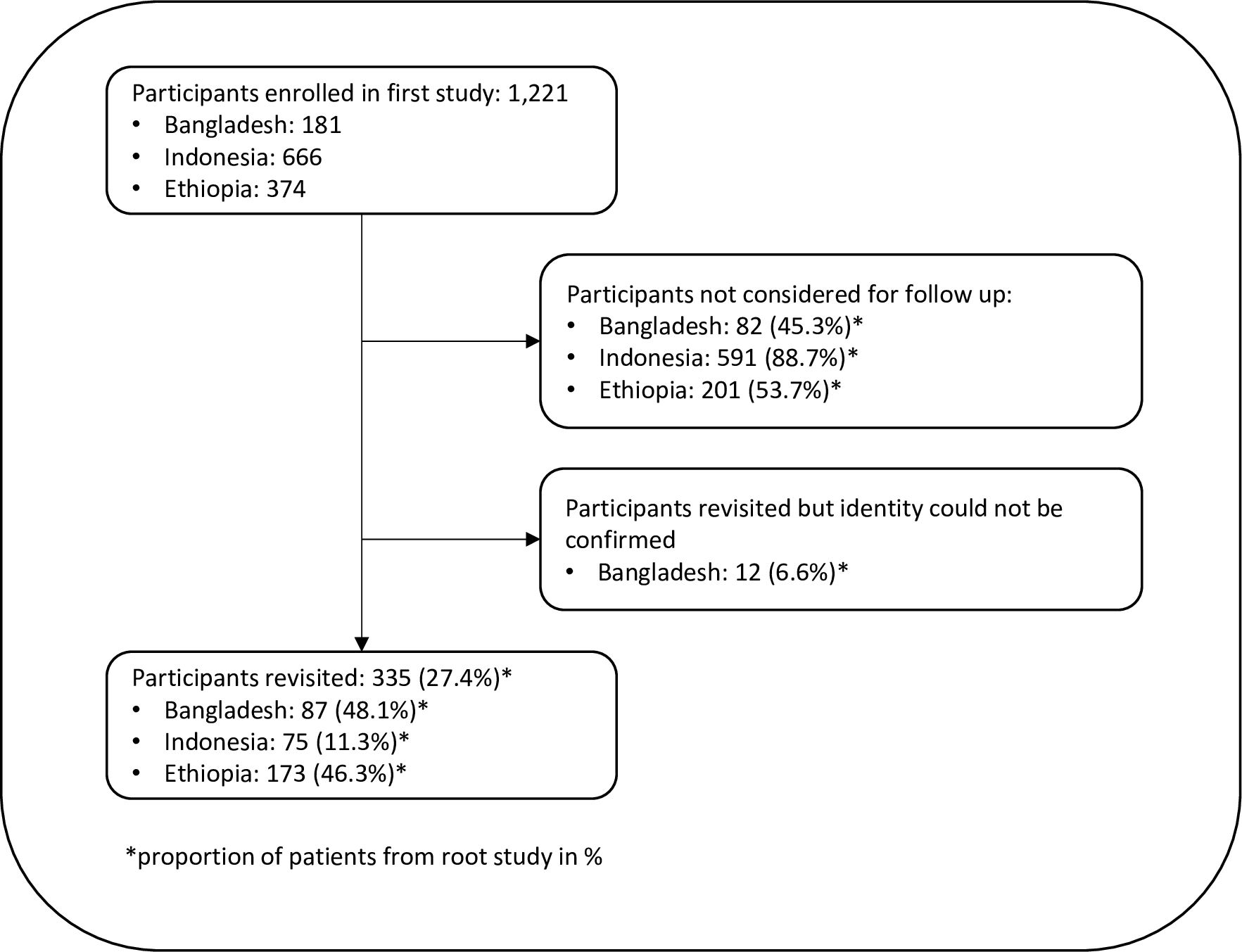

Supplement: S1 Fig — (TIF) [file pntd.0010406.s006.tif]

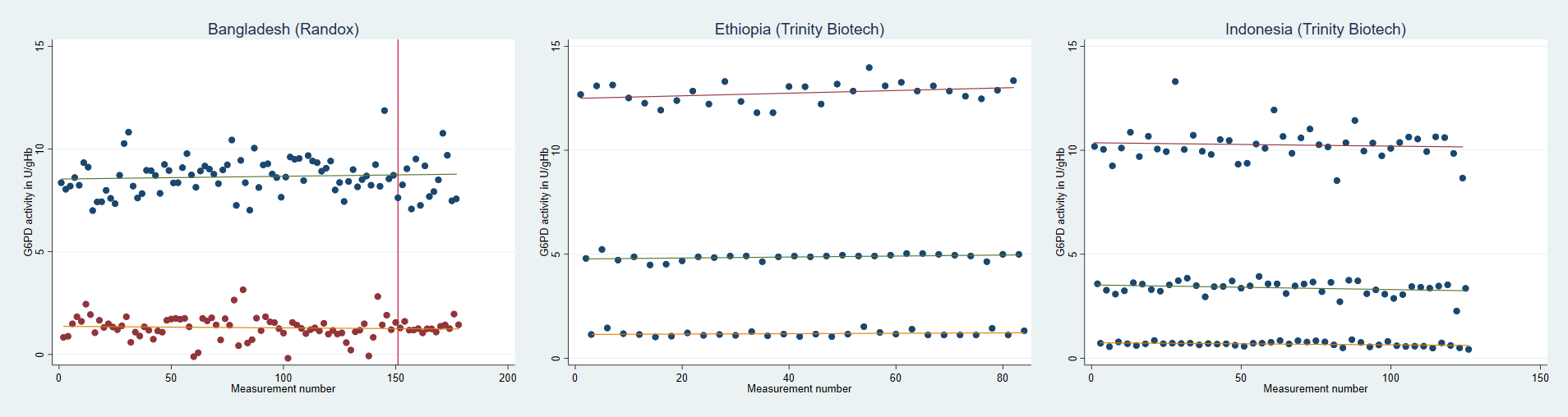

Supplement: S2 Fig — Legend: top horizontal line: line of best fit for G6PD normal controls, center horizontal line: line of best fit for G6PD intermediate controls (not done in Bangladesh), lowest horizontal line: line of best fit for G6PD deficient controls; vertical line (Bangladesh) follow up period where no testing was done (TIF) [file pntd.0010406.s007.tif]

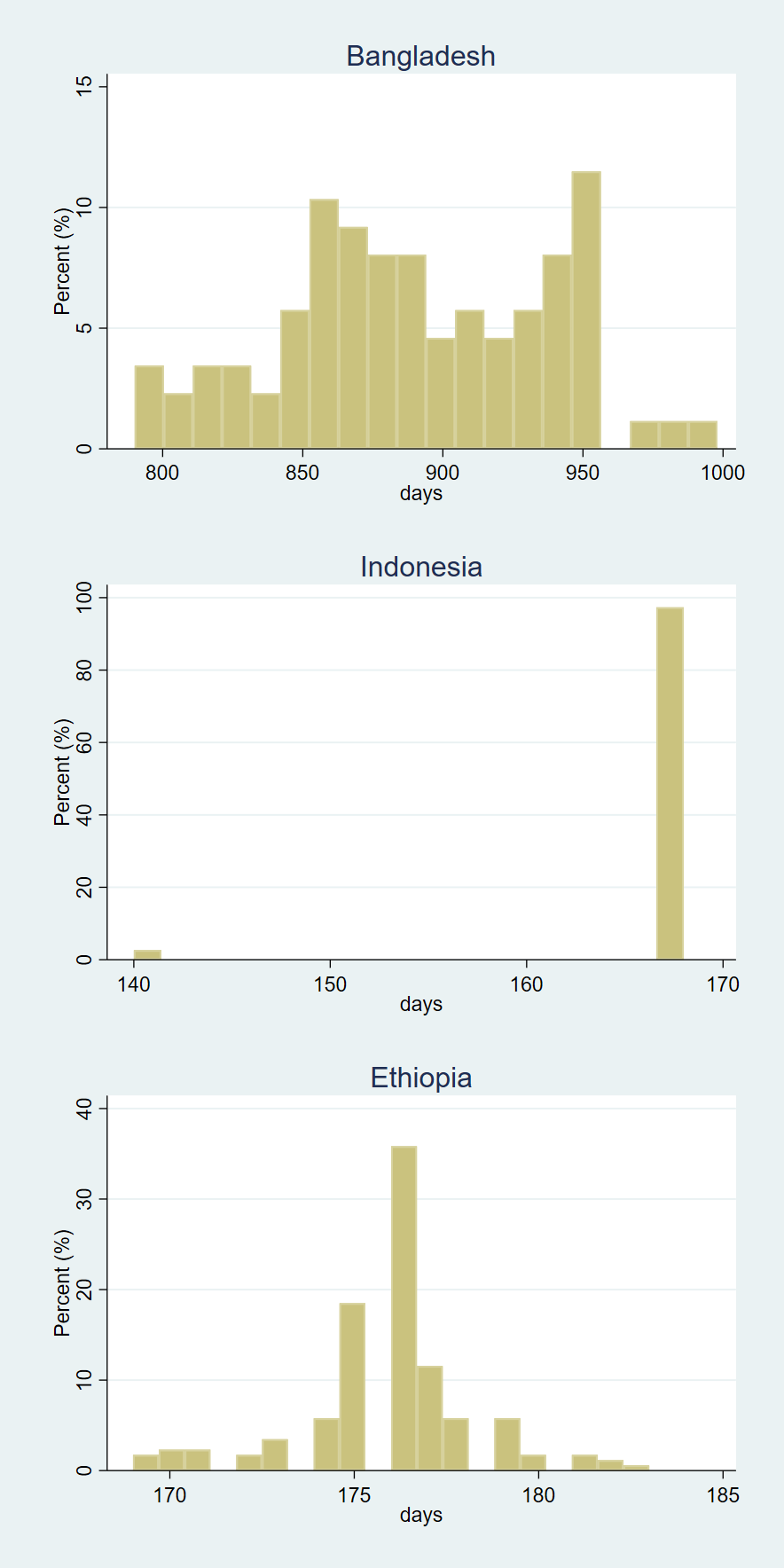

Supplement: S3 Fig — (TIF) [file pntd.0010406.s008.tif]
