# Supplementary material for: Variation in Glucose-6-Phosphate Dehydrogenase activity following acute malaria
Source: PLoS Negl Trop Dis. 2022 May 11;16(5):e0010406. doi: 10.1371/journal.pntd.0010406 (PMC9094517; doi:10.1371/journal.pntd.0010406)
Supplement: Attachment — Submitted filename: Rebuttal_v0.5.docx [file pntd.0010406.s010.docx]

**General comments**

- We have revised the whole manuscript for language, clarity, and word count.
- We realized there was a discrepancy in patient identifiers among some participants from Bangladesh, where the follow up visit was more than 2.5 years after enrolment. To clarify we went back to these participants to reconfirm their identity but could not reach 12 / 99 participants from Bangladesh and have excluded these from the analysis. Exclusion does not affect the overall outcome of this study.

**Methods**

**Reviewer #1:**

**“A subset of patients enrolled in the original trials were reviewed again 6 to 33 months after enrolment.” This statement is rather vague. Please indicate clearly how participants in the study were selected. Please provide a “consort chart” illustrating how the study participants were assembled? It may be necessary to provide separate charts for each site.**

**Please state clearly inclusion/exclusion criteria? If the criteria differ between sites, please provide a table with the criteria for each site?**

**“Since participants in Bangladesh were selected purposively …” I can’t follow this argument – please explain?**

We agree that sample bias is a potential confounding factor. The study was an opportunistic review of patients previously enrolled into clinical trials who could be found and reconsented for further follow up. In Indonesia and Ethiopia all patients during a specified time period were eligible and approached – and all consenting patients who could be identified were recruited. In Bangladesh a sub selection of 99 patients were approached (87 enrolled) with preference given to those with the lowest G6PD enzyme activity. The timing of follow up was opportunistic, with the only restriction that samples were collected at least 3 months after initial enrolment in the clinical trial to ensure samples were after haematological recovery from malaria.

To clarify this, we have added further details in Table S2 on the sample selection process for each study site. We have also provided a new (figure S1) for the study profile to present patients who were screened and enrolled into the original trials and then selected into the current longitudinal study.

We have also revised the text for further clarification:

Lines 116 – 122: *“A subset of patients enrolled into the original trials, who had been tested for G6PD activity, were contacted and those consenting to further investigation were enrolled into the longitudinal study. In Indonesia and Ethiopia all eligible individuals were approached. For logistical reasons, in Bangladesh a sample of 100 individuals (55%) with the lowest G6PD activity at baseline were selected, and patients who could not be contacted or declined to participate were replaced. Patients consenting to further investigation had a venous blood sample collected for repeat blood film examination or rapid diagnostic test for malaria and quantitative G6PD assay.”*

**Reviewer #2:**

**Methods; line 116: clarify better for the different studies how the subset were selected for the follow-up. In Ethiopia/Indonesia, was it just based on participant contactability? This matters given importance of the AMM estimates on the results. Also please clearly state the participants’ health status at the 2nd time point.**

The AMM was calculated as follows (lines 148 – 152): *“Since participants in Bangladesh were selected purposively 100% G6PD activity was defined as 7.03U/gHb based on results from a previous cross-sectional survey conducted in the same area using the same laboratory methods (20). In Indonesia and Ethiopia 100% G6PD activity was defined as the AMM based on the follow up measurements.”*

We have further clarified the health status of participants at follow up (lines 122 – 124): *“Patients with peripheral parasitaemia by microscopy or RDT as well as patients with self-reported history of malaria within the last 90 days, or major blood loss were excluded (S2 Table, S1 Figure).”*

**To avoid misinterpretation, it would be good to emphasise that the rates of deficiency seen at the two time points are based on selected sample groups: “among selected patients” (****only G6PD normal indivs recruited in Ethiopia/Indonesia at first timepoint; G6PD deficient patients preferentially included in Bangladesh). E.g. line 57 in Abstract, Fig 1 legend, Line 243 “selectively enrolled”.**

We agree that the reported prevalence of G6PD deficiencies refer only to the subgroup of individuals selected in our study. Whilst this has internal validity for comparing pre and post G6PD status it cannot be used to infer external prevalence of G6PD deficiency in the respective populations.

We have revised the abstract, the respective sentence now reads: (lines 51 – 55): *“Among selected participants (deficient participants preferentially enrolled in Bangladesh but not at other sites) G6PD activity fell between malaria and follow up by 79.1% (95%CI: 40.4 to 117.8) in 6 participants classified as deficient (<30% activity), 43.7% (95%CI: 34.2 to 53.1) in 39 individuals with intermediate activity (30% to <70%), and by 4.5% (95%CI: 1.4 to 7.6) in 290 G6PD normal (≥70%) participants.”*

We have changed the legend of figure 2 (former figure 1), which now reads (lines 229 – 230): *“Figure 2: Box and Whiskers plot of median normalized G6PD activity during malaria and follow up per study site among selected participants.”*

We have changed the discussion, which now reads: (lines 266 – 268): *“Among selected participants the greatest change in G6PD activity was observed in Bangladesh where a large proportion of deficient and intermediate individuals were included in the cohort, followed by Indonesia.”*

**Reviewer #3:**

**The design is not ideal to address the stated objectives as there are no control cases in the study.**

**The statistics presented are misleading and in some places inappropriately used to substantiate the authors conclusions**

We agree that a complementary control cohort of patients without malaria would have provided important information on the consistency of G6PD activity in individuals over time independent of malaria. However, this was not feasible within our study design. These longitudinal cohorts of aparasitaemic individuals are being addressed in subsequent clinical trials (preliminary results suggest changes are far less marked in those without malaria). To acknowledge this limitation, we have revised the discussion as follows (lines 326 – 330): “*Our study has several important limitations. First, the study adopted an opportunistic design, revisiting individuals who had been enrolled into previous antimalarial clinical trials. Since only patients initially presenting with malaria were enrolled and there were no aparasitemic controls, one cannot definitively infer that initial elevations in G6PD activity were attributable to the initial peripheral parasitaemia.”*

**Results**

**Reviewer #1:**

**Table 1: please explain what you mean by “unselected cohort”? I assume you mean excluded?**

We have changed the headings in table 1 that now read: “*Excluded cohort*” and “*Selected cohort*” (line 188).

**Please highlight this excluded cohort in the consort chart?**

Excluded and selected cohort are compared in table 1. We have also added a consort chart (figure S2). We did not collect the reason for non-participation so cannot provide additional information in the consort chart.

**“The time between presentation with malaria and follow up ranged between 790 and 998 days in Bangladesh, 140 and 168 days in Indonesia, and 169 and 183 days in Ethiopia.” Please provide a more detailed statistical description of the distribution of F/U dates.**

The timing for follow up varied significantly over prolonged periods of time. However, the key consistent factor was that all patients were sampled >3 months following initial period of malaria so had had complete haematological recovery.

We have revised the text on lines 191-193 to provide further details: *“**The median time between presentation with malaria and follow up varied significantly between sites: 887 days in Bangladesh (range: 790 to 998), 168 days in Indonesia (range: 140 – 168), and 176 days in Ethiopia (range: 169 – 183) (S2 Fig).”*

**Pls. consider providing a Figure to illustrate the distributions.**

We have added figure S3. Follow up occurred at least 4 months after initial malaria episode thus allowing time for complete haematological recovery and patients did not have detectable parasitaemia at the time of convalescent review. We have highlighted this in the discussion on limitations (lines 339 - 345): *“Thirdly, the time to follow up differed significantly between sites. Haematological recovery following acute malaria usually occurs within 28-63 days (38, 39), although after haemolytic events it can take significantly longer for the age distribution of RBCs to return to steady state (40). The primary objective of the current study was to resample patients following complete haematological recovery and during a period when the patients were aparasitaemic with no recent history of major blood loss or malaria within the previous 3 months, and this was achieved in all study sites with a minimum time to resampling of 140 days.”*

**“The greatest difference between acute presentation and follow up was observed in Bangladesh” not only was this difference greatest in Bangladesh. The time window between 1st and 2nd measurement was also by the widest. Perhaps the extended duration explains the differences in enzyme activity? Pls. explain why you think this is not the case?**

As stated above the key feature here is sampling patients at a time of health and complete haematological recovery from their acute malaria. Hence the fact that the timing of sampling varied whilst significant, is less relevant. However, we acknowledge this as a potential limitation and have revised the discussion accordingly (kindly see the comment above (lines 339 – 345).

**“Figure 2: Change in G6PD activity between malaria and follow up, stratified by G6PD status during follow up” Pls. rephrase the title? … between the malaria episode and follow up, stratified by G6PD status at the time of follow up**

We have revised the title of figure 1 (former figure 2) as suggested (lines 210 - 211).

**Pls consider a similar analysis as presented in Fig 2 instead of stratified G6D status stratified by site? (at a minimum include table S5 in the main text.)**

The primary analysis is a comparison of the change in G6PD activity between deficient, intermediate and normal status. Since enzyme activity is normalised at each site (using local adjusted male median values), it is more informative to pool these data across sites and present stratified by G6PD status.

To show the data the reviewer requests we have added categories by site in Table 2. The difference is most apparent in Bangladesh, although the number of individuals with intermediate or deficient G6PD activity in Indonesia and Ethiopia, preclude comparison.

**“****The estimated mean change in Hb associated with an increase of 10 % units in G6PD activity was -0.05 g/dL (95% CI: -0.14 to 0.05, p=0.336) at enrolment and -0.03 g/dL (95% CI: -0.11 to 0.06, p=0.560) at follow up.” This observation could be interesting but seems to be difficult to understand the way it is presented. The change in enzyme activity is over the time period from malaria episode to F/U date? Why don’t you report the Hb change over the same period?**

The aim of this analysis was to assess whether drug induced haemolysis was dependent upon G6PD activity as measured during presentation with infection or dependent upon G6PD activity when measured during steady state. The assumption was that G6PD activity measured during follow up was reflective of G6PD activity at steady state. To clarify we have provided additional details in the methods section where it now reads (lines 165 – 173):

*“It was assumed that in the absence of haemolysis or major blood loss, G6PD activity does not vary significantly over time, and therefore G6PD activity during follow up reflects an individual’s G6PD activity at steady state. As a proxy for drug induced haemolysis the change in Hb between the day when Pq radical cure was first administered and seven days later was considered. Linear regression analysis was used to determine whether activity during steady state or the G6PD activity during the malaria episode was predictive of drug induced haemolysis. Hb concentration on day 7 was the dependent variable and G6PD activity during malaria and follow up were considered exposures after controlling for sex, parasite density, and Hb at treatment start [10, 21]. Since day 7 Hb was not recorded for Indonesian patients this cohort was excluded from this analysis.”*

The corresponding results section now reads (lines 250 - 255): *“In total 30 patients from Bangladesh and 172 patients from Ethiopia had a Hb concentration measured at enrolment and seven days after starting PQ based radical cure. Their Hb dropped by a mean of 0.1 g/dL (95%CI: -0.3 to 0.0) in the first seven days of treatment. A 10% increase in G6PD activity at enrolment was associated with a drop in day 7 mean Hb of -0.04 g/dL (95%CI: -0.13 to 0.05, p=0.471) and a 10% increase in G6PD activity during steady state was associated with a decrease of 0.02 g/dL (95%CI: -0.11 to 0.06, p=0.611).”*

**Reviewer #2:**

**The implications of the results are most significant for P. vivax patients. Would there be statistical power to allow a subanalysis with only Pv patients?**

We have added table 2 that presents the observed change in activity / species (line 221) and have added table 3 where we compare the shift in G6PD categories by species (line 247).

**Supplementary figure 1: suggest including control recommended ranges to help interpret the variation seen. Could also add control manufacturer names to plot titles.**

We have added the manufacturer names to S2 Figure, however were unable to add recommended ranges as these are lot number specific and we were unable to retrieve these data.

**Reviewer #3:**

**The results are clearly presented but the analysis is misleading A major limitation in the analysis is the fact that the analysis (change in G6PD activity) per Plasmodium species is not presented, and the fact that the one site with only P.vivax infections does not support their conclusions is not discussed.**

Whilst we acknowledge limitations of the study, the primary objective of the study was to explore the effect of parasite induced haemolysis (irrespective of species) on G6PD enzyme activity. However, to address the reviews concerns we now present the percentage change in G6PD activity according to species (see response to Reviewer #2). The highly significant shift in categories remain apparent for both patients with *P. falciparum* and *P. vivax* infections.

**Conclusions**

**Reviewer #1:**

**“Activity increased by 11% overall, ranging by almost 80% in deficient individuals, 45% in intermediate individuals, and 5% in individuals categorized as G6PD normal during follow up.” This sentence can be interpreted in different ways. Either the enzyme activity increased during follow up or the individuals were categorized as normal during follow up. Please consider rewriting to provide more clarity?**

These percentage shifts refer to the changes within an individual’s activity acutely and during follow up. We have revised to the text to make this clearer.

*Lines 203 - 204: When comparing the same individual’s G6PD activity during malaria and again during follow up, enzyme activity was -10.4% (95% confidence interval [95%CI]: -13.9 to -6.9) lower during follow up.”*

**“during health” is poor English – pls. rephrase?**

We have revised the text accordingly (line 286): *“The observation that G6PD activity increases during acute malaria is not unexpected,…”*

We have changed lines 303 – 305, which now reads: *“Our findings raise the possibility that aparasitemic individuals identified as severely or intermediate G6PD deficient may not necessarily be at high risk of drug induced haemolysis during malaria.”*

**“Over 80% of study participants diagnosed with less than 70% activity during follow up would have been denied primaquine radical cure if they had not been re-tested.” In this sentence the temporal relationship between the first test and the second test is the wrong way around. Fewer readers may be confused if you would consider rephrasing? “Over 80% of study participants diagnosed with less than 70% enzyme activity during follow up would have been denied the radical cure if health care providers would rely solely on the follow-up test instead of the test result at the time of the acute malaria episode”.**

We have now restated that individuals were categorised as being deficient, intermediate or normal according to the local AMM and their enzyme activity during follow up (ie without malaria).

Line 200 - 202: *“Based on results from a previous cross sectional study in the same population 100% G6PD enzyme activity was defined as 7.03 U/gHb in Bangladesh [22], and based on the G6PD measurements during follow up 100% activity was defined as 9.24U/gHb in Indonesia, and 12.13 U/gHb in Ethiopia (Table 1).”*

Line 260 - 264: *“Overall an individual’s G6PD activity was 10% greater when measured during an acute episode of malaria compared to when remeasured during follow up. When G6PD status was categorized according to an individual’s enzyme activity during follow up, two thirds of participants with severe deficiency and almost 90% of those with intermediate deficiency had normal enzyme activity (>70%) at the time of presentation with malaria.”*

**“Reassuringly quality control procedures suggested reliable testing throughout the study period at all three sites. ‘ perhaps the keyword here is “consistent” rather than “reliable”?**

We have changed lines 332 – 333, which now reads: *“Reassuringly quality control procedures suggested consistent testing throughout the study period at all three sites”*

**Please discuss the advantages/disadvantages of combining data from different sites when the data are so different. Isn’t that comparing “apples with oranges”?**

We have added a section in the discussion on this (lines 354 - 360): *“Our study is confounded by heterogeneity between study sites, selection bias (specifically in Bangladesh), differences in prevalence of G6PD deficiency, and potentially known local variants. Despite this limitation, the changes in G6PD activity were apparent in varying degrees across all three sites and highlight that a substantial proportion of individuals with severe and intermediate G6PD deficiency were diagnosed as G6PD normal when presenting with acute malaria. Subsequent prospective case-control studies, with molecular confirmation of genetic variants, are warranted to confirm and quantify these findings in other endemic settings.”*

**“Our findings have important public health implications for identifying populations at risk of haemolysis, as well as test and treat strategies for radical cure.” This sentence is meaningless – why waste the time of the reader? Please clearly state the implications!**

Thank you for highlighting this omission. We have revised the final conclusion to clarify the potential public health importance of our findings, but also acknowledge that further studies are needed to confirm these (lines 361 – 367): *“In conclusion, our study highlights that an individual’s G6PD activity is significantly higher during acute malaria, particularly those with intermediate or severe G6PD deficiency. If these findings are confirmed, it raises the possibility that an individuals’ G6PD status is not static, and thus the risk of drug induced haemolysis when presenting with malaria cannot be assumed from measuring an individual’s enzyme activity when aparasitaemic. From a public health perspective, the population attributable risks of drug induced haemolysis may be significantly lower than that predicted from cross sectional surveys.“*

**Reviewer #3:**

**The conclusions are not supported by the data presented.**

**The limitations are not clearly described**

We acknowledge some of the Reviewers concerns and some important limitations of our study. We have now provided a far more detailed description of these limitations (lines 326 – 353). We also provide further subgroup analysis highlighting that the shifts in G6PD enzyme activity are i) apparent at all sites (despite low numbers of G6PD deficient individuals in Indonesia and Ethiopia) and ii) present in patients with either *P. falciparum* or *P. vivax* malaria. Despite these limitations we believe that our findings are important and are needed to stimulate further prospective studies on this important subject.

**Editorial and Data Presentation Modifications**

**Reviewer #1: Abstract**

**“Patients with uncomplicated malaria were recruited” should be Patients with uncomplicated vivax malaria …**

We enrolled malaria patients with all species into the trial. We have revised the text to clarify this (line 49): *“Selected patients with uncomplicated malaria were recruited…”* and (line 58 – 59): *“The change in G6PD activity was apparent in patients presenting with either P. vivax or P. falciparum infection”*

**“At the time of follow up” … please indicate the median follow-up time?**

We have added the following sentence to the abstract (lines 50 – 51): *“…;G6PD activity was measured by spectrophotometry at the initial presentation with malaria and again a median of 176 days later (range 140 to 998) in the absence of malaria “*

**“… this may have implications for risk of drug induced haemolysis.” Many readers focus on the conclusions provided in the abstract. The conclusions as they are currently written are not helpful. Please indicate which implications do you have in mind? Are they good, bad, or neutral? You write in the discussion “in patients presenting with malaria the prevalence of severe and intermediate G6PD deficiency is considerably lower than that predicted from cross sectional surveys” which frequently guide prescription practices. Your study suggests that these prescription practices may be overly cautious. Please dare to be controversial.**

We have rephrased the conclusion which now reads (lines 62 – 65): *“These findings suggest that G6PD activity rises significantly and at clinically relevant levels during acute malaria. Prospective case-control studies are warranted to confirm the degree to which the predicted population attributable risks of drug induced haemolysis is lower than would be predicted from cross sectional surveys. “*

**Summary and General Comments**

**Reviewer #2:**

**This is a very worthwhile study that carries important implications for assessing population-level P. vivax treatment risks. The manuscript is clearly presented, and the interpretation of results a good representation of the presented data.**

**Patients’ genetic variants were not characterised. If available, this would have been valuable additional data to help interpret the observations: authors could perhaps mention this in their discussion. It matters because the global diversity of G6PD mutations means that the trend observed in this study from these 3 sites may not be replicated everywhere. E.g. would this same difference be visible in patients with the Mediterranean mutation? Or is the effect less pronounced in patients where even young RBCs have low G6PD activity?**

We agree that this is a significant limitation and added included this in (lines 346-353*): “Fourth our analysis did not include genetic confirmation of the diagnosis of individuals with G6PD deficiency. More than 230 clinically relevant G6PD variants have been described [8, 41] that confer different degrees of enzymatic deficiency. The observed changes in enzyme activity associated with acute haemolysis may differ between G6PD variants. For example, for individuals with the Mediterranean variant almost all RBCs, including young cells, are severely deficient and thus parasite or drug induced haemolysis will have a minimal impact on the overall activity, whereas for individuals with milder variants, G6PD activity would be more likely to normalize. [42, 43]. Hence observed changes in enzyme activity may differ significantly by region.“*

**Minor comments:**

**Line 50: “replacing”: implies direct causation, suggest “leading to replacement of…”**

We have rephrased the entire abstract in order to address the reviewers’ comments and remain within the 300-word limit. The mentioned sentence is no longer included.

**Lines 87-90: for clarity, suggest listing references mid-sentence.**

We have replaced both references by a more recent one from the same author Lucio Luzzatto:

8.	Luzzatto L, Ally M, Notaro R. Glucose-6-Phosphate Dehydrogenase Deficiency. Blood. 2020.

**Line 112: not sure S2 table is right one to link to here?**

S2 Table lists the site-specific laboratory procedures and is referenced at the end of the paragraph that covers the laboratory procedures.

**Line 164: day 7 testing not previously mentioned; Methods implies all testing was on day 0.**

The respective sentence is now at line 144 - 145. To clarify we have added the following sentence earlier in the manuscript: *“Data collected at enrolment were extracted from existing databases, including sex, age, G6PD activity, Hb concentration (at baseline and day 7), and species of infection.”*

**Line 170: typo: “Norther Territory”**

Corrected in line 176

**Line 292: “support previous model ?recommendations”?**

The sentence now reads (lines 293 – 296): *“Our findings also support previous models proposing that a delay in starting hypnozoitocidal treatment until the end of schizontocidal treatment (once the most deficient RBCs have been lysed), could reduce drug induced haemolysis (31).”*

We now reference a study by Watson et al (2017), who modelled the safety of a step wise increase in daily primaquine doses in G6PD deficient individuals. The authors specifically point out that this model needs to be confirmed in patients.

**Line 286 and 301: check wording**

Line 286: We have changed the respective sentence to (lines 313 – 315): *“In a recent study from Cambodia, G6PD activity was measured over an 8-week period in patients with P. vivax malaria treated with weekly PQ (33). G6PD activity increased between the first and second PQ dose, with the greatest change in activity observed in deficient patients.”*

Line 301: The respective sentence reads (lines 337 – 339): *“However, after adjusting for Hb concentration in the multivariable analysis the estimated changes in G6PD activity levels between the acute malaria episode and follow-up were still apparent. “*

**Reviewer #3:**

**In this article the authors present very interesting data on the difference in G6PD activity in individuals when malaria positive versus when malaria negative. The data is interesting and the sheer number of cases for which they have this matched data could be useful. There are however some deficiencies in the data that need to be clarified and perhaps some issues in the manner in which the data is presented that should be resolved. The presentation of the statistics seems to be somewhat leading and possibly not necessarily supported by the data presented.**

**Major limitation of the study design:**

**The study recruited the patients as malaria positive and then conducted a follow-up visit with a wide ranging period of time between the two visits. The study does not have a control arm of healthy individuals at the time of recruitment and time of follow-up. This is significant because all of the significant changes in G6PD status arise from the one site, Bangladesh, which noticeably (i) had the widest time gap between malaria and follow-up visits and (ii) was the only site to use the Randox assay and (iii) it is the one site where “ In Bangladesh participants were chosen to include the fraction with lowest G6PD activities during enrolment” so these will have had most samples that were on or close to the G6PD deficient/intermediate/normal thresholds. A lot of the differences may arise from slight differences in the Randox assay lots, the individuals/details of how the tests were run or differences in how the specimens were handled which would not be identified by the test controls. The AMM from the follow-up visits were used to determine the percent activity across both malaria and follow-up visits, so any bias resulting for the time lag would accentuate those observed. Without a control arm this cannot be resolved. These limitations should be specifically called out as they represent a significant limitation to the analysis.**

**Noticeably the one site, Ethiopia where only P.vivax cases were collected showed the median G6PD activity increases in the follow-up. Which leads to another major limitation with regards to how the data is presented. The authors make their claims based on combined P.vivax and P.falciparum infections while their discussion is only relevant to P.vivax infections. It is surprising that the authors do not present the data disaggregated by species. Specially as the inherent (and not mentioned) assumption that both species have the same hematological effects is not addressed. The 5 samples that are G6PD deficient in the follow-up and not during the malaria infection, were all from Bangladesh and presumably (based on the discussion) in P.falciparum infections.**

We thank the reviewer for their frank criticisms and acknowledge many of the shortcomings of our opportunistic study. We have revised the whole manuscript to provide additional material and analyses, including an analysis stratified by species, and also clearly state the limitations in the discussion.

Despite biases between sites and confounding of *P. falciparum* and *P. vivax* malaria, our findings are robust. We see significant shifts in enzyme activity at all three locations (admittedly significantly higher in Bangladesh than Indonesia or Ethiopia), as well as with patients presenting with *P. falciparum* or *P. vivax*.

We believe that our findings have potentially very important implications for public health but acknowledge that further prospective studies with molecular analyses are needed to make firm conclusions. These are ongoing and we hope that other researchers will conduct similar studies in other locations.

Specific comments include the follow:

**1.	The issue that there is no control arm for in this study should be clearly called out as it is a major limitation given the reasons mentioned above**

We have added this as the first limitation in the discussion (lines 326 – 330): *“First, the study adopted an opportunistic design, revisiting individuals who had been enrolled into previous antimalarial clinical trials. Since only patients initially presenting with malaria were enrolled and there were no aparasitemic controls, one cannot definitively infer that initial elevations in G6PD activity were attributable to the initial peripheral parasitaemia.”*

**2.	The authors should present the change in G6PD activity disaggregated by species, P.falciparum and P.vivax and discuss the hemolytic risks according to the P.vivax results.**

Also raised by reviewer #2. We have revised the text in the methods, results and discussion to explore the shift in enzyme activity separately for *P. falciparum* and *P. vivax*. The significant changes in G6PD activity are apparent for both species and are now presented in tables 2 and 3.

**3.	In the abstract and discussion the authors should clarify that the significant drop in G6PD activity leading to different classifications almost entirely arise from the Bangladesh data (which has the above mentioned biases and limitations) and not from all three sites. The discussion should explain why the trend is only observed at one of the three sites, and specifically not observed in the site with only P.vivax infections (Ethiopia). The way the data is presented is misleading in that it suggests that this an observation across all sites.**

We agree this is important, although the trends in overall enzyme activity are apparent in both Bangladesh and Indonesia. Power in shifting categories in Ethiopia and Indonesia is reduced due to low prevalence of deficiency in these sites. We have revised the text as follows in the results (lines 234 – 237): *“The country specific drop in activity corresponded to the proportion of participants with decreased activities enrolled. In Bangladesh 33 (37.9%) individuals were categorized with intermediate G6PD deficiency and 6 (6.9%) with severe deficiency. The corresponding proportions were significantly lower in Indonesia (6.7% and 0.0%) and Ethiopia (0.6% and 0.0% respectively).”*

We also highlight this in the discussion where it now says (lines 266 – 272): “*Among selected participants the greatest change in G6PD activity was observed in Bangladesh where a large proportion of deficient and intermediate individuals were included in the cohort, followed by Indonesia. In Ethiopia only one individual had reduced G6PD activity during follow up, a 25-year-old male with 61% enzyme activity during malaria and 62% during follow up. Whilst the overall mean G6PD activity in Ethiopian participants was 4% higher during follow up compared to during the malaria episode, this modest rise was not clinically relevant.”*

**4.	Abstract lines 61 to 63: “67% (4/6) of deficient and 89% (36/41) of…” is an example of misleading statistics. Most of these cases (the 4 and 36) arise from the Bangladesh study in which all except one deficient and 4 intermediates were normal and so they could only go one way in the follow up visit. The Ethiopia and Indonesia study included all normal by FST so again this sample set was bias to only have switches towards intermediate or deficient cases.**

Following this and previous suggestions we have rephrased the abstract. The relevant sections now read (lines 55 – 58): *“In Bangladesh and Indonesia G6PD activity was significantly higher during acute malaria than when the same individuals were retested during follow up (40.9% (95%CI: 33.4 - 48.1) and 7.4% (95%CI: 0.2 to 14.6) respectively), whereas in Ethiopia G6PD activity was 3.6% (95%CI: -1.0 to -6.1) lower during acute malaria. “*

**5.	Author summary lines 40-42: “ …85% (40 / 47) of individuals ineligible for standard treatment during follow up, were eligible for treatment during malaria.” This is again misleading (assuming all the other issues are OK) since actually the 36 with 30-70% G6PD activity are eligible in most countries to 14 day primaquine.**

We agree and have removed any reference to primaquine treatment specifically. The conclusion now reads (lines 40 – 42): *“The increase in G6PD activity during malaria is probably triggered by the Plasmodium infection, the observed change in G6PD activity may alter the risk profile of standard malaria treatment with 8-aminoquinolines.”*

**6.	As per the above statement in the discussion lines 279-280, 70% is not the threshold for withholding primaquine, only those with G6PD activity below 30% would have primaquine withheld according to WHO guidelines.**

We agree with the reviewer and have removed this statement.

**7.	Line 282: “All three participants with lower G6PD activities during malaria compared to follow up,…” this seems to be misleading since their supplemental data and their Ethiopia data (Figure 1) which is relevant since these are all P.vivax cases there are than 3 cases lower G6PD activities compared to follow up.**

This was an error, we were referring to G6PD categories. The corrected sentence now reads (lines 309 – 312): *“**All three participants with normal G6PD activities during malaria and intermediate activities during follow up, had borderline G6PD activity (60% to 70%) at the first measurement and the mean increase in G6PD activity during follow up was less than 20%.”*
