# Supplementary material for: Variation in Glucose-6-Phosphate Dehydrogenase activity following acute malaria
Source: PLoS Negl Trop Dis. 2022 May 11;16(5):e0010406. doi: 10.1371/journal.pntd.0010406 (PMC9094517; doi:10.1371/journal.pntd.0010406)
Supplement: Attachment — Submitted filename: Rebuttal2_v0.4.docx [file pntd.0010406.s011.docx]

**We would like to thank the reviewers and the editor for their constructive feedback.**

**Reviewer #1 and #2 have accepted our previous rebuttal and we respond to the additional comments made by reviewer 3 below:**

**Introduced changes not requested**

- We have corrected the Y -axes in figure 1, which now has a standardised Y axis.
- Reviewer 3’s main concern is that our findings are driven predominantly by the Bangladesh data, and that the greater differences at low enzyme activity reflect a regression to the mean (rtm). To address this, we have calculated the rtm effect and found the observed effect exceeds the calculated rtm effect. We have added details on the procedures in the methods (lines 162 – 165) and in the results:

“*The calculated rtm effect for deficient individuals was -60.9% (95%CI: –71.4 to -50.5, p<0.001) at the 30% activity threshold, and -31.4% (95%CI: -36.8 to -25.9, p<0.001) at the 70% threshold*.” (lines 225 – 226).

We discuss these findings further in the limitations section:

“*…Finally, the observed difference in activities could have arisen from regression to the mean, with extreme values measured during follow up becoming less extreme when measured again during acute malaria [21]. However, at both the 30% and 70% definitions for G6PD deficiency the calculated rtm effect was below the observed effect. Keeping the small sample size of deficient individuals in mind, this suggests a true trend.*” (lines 368 – 372)

**Comments from reviewer 3:**

**1.	Bangladesh data set: There are several concerns with this data set and how it is presented. The follow-up data set has a median (and mean) G6PD value approximately 2.5 U/gHb lower than the acute malaria set and almost 2.0 U/g Hb lower than that of 100% normal used to determine the percent change in activity. In contrast the malaria and healthy G6PD medians are within 0.5 U/g Hb of each other and equally close to that of the authors 100% normal. This has several implications: (i) the Bangladesh data set is an outlier compared to the other two, and extremely unusual, (ii) using a 100% normal G6PD activity level that is 2 U/gHb or 40% greater than the population median does not make, by definition, any sense and will only exacerbate any difference with the acute malaria cases where the median was above the 100% normal, (iii) the bias generated by this will significantly and disproportionately affect any pooled analysis by species as presented by the authors in Table 2.**

The participants in Bangladesh were selected purposively to enrol patients with the lowest G6PD activities, hence defining 100% activity from this subset is incorrect and would have introduced its own bias. Instead, we followed standard practice and defined the Adjust Male Median from a cross sectional survey conducted in the same population applying the same laboratory methods [1]. The prevalence of deficient and intermediate participants was 9% and 13% respectively in the cross-sectional survey, in contrast corresponding figures were 7% and 38% in the selected subset.

i) We agree that the Bangladesh dataset is an outlier. The prevalence of intermediate/severe deficiency participants after recovery was 45% compared to 1% in Ethiopia and 7% in Indonesia. We hypothesize that the greatest shift in G6PD activity between acute malaria and convalescence occurs in participants with a reduced G6PD activity who are more vulnerable to parasite induced haemolysis. This provides a biologically plausible explanation that the shifts are greatest in Bangladesh. Country specific changes are now presented in Table 2 (line 228). And we have clarified this in Lines 373-375 regarding limitations

*“**Our study is confounded by heterogeneity between study sites, selection bias (specifically in Bangladesh), differences in prevalence of G6PD deficiency, potentially known local variants, and potential regression to the mean.”*

ii) Calculating 100% activity from a purposively selected subset is not appropriate and would have introduced bias. Calculating the adjusted male median activity from follow up readings (5.16 U/gHb) as done for the other sites would be confounded by selection of more deficient participants, the observed effect in percent would have been even more extreme. We therefore used the standard practice of deriving the adjusted male median from a cross sectional survey (7.03 U/gHb)[2] done within the same population and using the same spectrophotometry methods. This approach is actually more conservative than that proposed by the reviewer.

iii) We have amended table 2 (line 228) to present the results stratified by species and country.

**2.	Bangladesh data set: The authors only included G6PD deficient cases identified during the follow-up visit, and no deficient cases identified during the acute malaria phase, so inherently any analysis of the deficient cases will be biased and misleading. As the authors also point out, none of these were confirmed by sequencing, so it is just as likely that these are an artefact of the low G6PD values generated by the reference assay during this period. The controls seem to work well so possibly there was a specimen integrity issue. An indication that this sample set is strange is the data in supplementary table 5 with more “intermediate” males than females.**

In the original Bangladesh clinical trial there were 175 participants of whom only 1 had G6PD activity <30% and 5 had intermediate activities (30-70%). We re-enrolled the severely deficient case and 4 of the 5 intermediate cases.

We acknowledge that some shift in the recorded G6PD activity could be attributable to sample processing and assay variation, as reported previously [3]. However, the trend to consistently lower activities at convalescence is consistent across Bangladesh and Indonesia. In Ethiopia the prevalence of deficiency is much lower, only 1 of the participants had a low enzyme activity and thus the power to detect a shift was not apparent.

We agree that the lack of G6PD genotyping is a limitation and acknowledge this on Lines 360-361:

*“Fourth our analysis did not include genetic confirmation of the diagnosis of individuals with G6PD deficiency.”*

We acknowledge the lack of a control group in Lines 341-344:

*“Since only patients initially presenting with malaria were enrolled and there were no aparasitemic controls, one cannot definitively infer that initial elevations in G6PD activity were attributable to the initial peripheral parasitaemia.”*

We reflect on the high number of males with intermediate activity on lines 334-339:

*“In our study 12% of males had intermediate G6PD activity (30%-70%) recorded during follow up (S5 Table); this was most apparent in Bangladesh where patients with lowest G6PD activities were purposively selected. The G6PD gene is located on the X-chromosome and males are thus either hemizygous deficient or normal [8], however intermediate activities have been reported from previous cross-sectional surveys [23, 38, 39] and may reflect the AMM (100% activity) being calculated from heterogenous populations with G6PD variants associated with different severity.”*

**3.	Indonesia and Ethiopia: Given the outlier characteristics of the Bangladesh data set it is worth (and even in absence of that) looking at the G6PD levels per species per site and Ethiopia and Indonesia pooled. When this is done, at least by median G6PD values there is no significant difference in G6PD values between acute and follow-up visits, maybe a slight decrease for Pf (in Indonesia) and slight increase for Pv (in BOTH Indonesia and Ethiopia). By presenting the data the way it is presented in Table 2, rather than by species by site, as it would typically be done in this kind of analysis, this is not shown.**

We have added the suggested additional categories to table 2 (line 228), to present the change in activity per species for the entire study population as well as stratified by study site.

In Ethiopia all participants except for one individual with intermediate activity (who did not change significantly) were G6PD normal throughout the study period and no overall change in activity was observed.

In contrast in Bangladesh and Indonesia the mean G6PD activities for both *P. falciparum* and *P. vivax* decreased between enrolment with malaria and follow up, the trend being more pronounced in Bangladesh.

We discuss these observations on Lines 275-281:

*“Among selected participants the greatest change in G6PD activity was observed in Bangladesh where a large proportion of deficient and intermediate individuals were included in the cohort, followed by Indonesia where two out four participants with intermediate activities had normal activities when revisited. In Ethiopia only one individual had reduced G6PD activity during follow up, a 25-year-old male with 61% enzyme activity during malaria and 62% during follow up. Whilst the overall mean G6PD activity in Ethiopian participants was 4% higher during follow up compared to during the malaria episode, this modest rise was not clinically relevant.”*

**4.	Indonesia and Ethiopia: When only looking at these two data sets, the only changes in status is for 6 individuals, 3 of which are males so actually no change in status, and all males and females would have received primaquine. These changes in status maybe attributed to variation in reference testing**

Whilst variation in reference testing may confound changes – the shifts in enzyme activity are consistent.

We have added the change in G6PD category per country to table 3 (line 255). In Ethiopia 1/172 participants had intermediate activities during enrolment and follow up, in Indonesia 4/75 participants had intermediate activities during enrolment of which 2 had normal activities during follow up.

We have revised the discussion accordingly on lines 275-281 (as stated in response to comment #3):

**5.	There is no biological or pharmacological sense in pooling the analysis across both species. Pv infects almost primarily reticulocytes and at lower parasite densities compared to Pf which infects a broader range of red blood cells and at higher parasite density. These differences are likely to have an impact on the relative effect of infection on G6PD activity levels, which their data (Indonesia and Ethiopia) actually seems to show. Notably (even in Bangladesh) all clinically relevant changes in G6PD activity are in Pf.**

We agree that the biology of red cell invasion differs between *P. vivax* and *P. falciparum*, however the basic principle remains similar, that parasite infection results in haemolysis of both infected and uninfected erythrocytes. Although peripheral parasitaemia tends to be significantly higher for Pf infection than Pv, the majority of red cell loss is attributable to indirect haemolysis, with loss of unparasitized cells accounting for 70-90% of the drop in Hb [4, 5]. Hence, we believe that there is a good rationale to pool all plasmodia infection, but to address the reviewers concerns also present changes in G6PD activity according to species.

We have revised Table 2 and now present changes in activity per species, stratified by country. In patients with intermediate or deficient activity during follow up the change was greater in patients with *P. vivax* compared to with *P. falciparum* (-49% vs. -39%, table 2, line 228). The trend is similar in Bangladesh and Indonesia (table 2).

We have revised the discussion to highlight this (lines 273-275):

*“The overall change in activity was more pronounced for patients with P. vivax infection, however the differences in an individual’s G6PD status were apparent in patients presenting with P. vivax and those presenting with P. falciparum infection.”.*

We have also revised the discussion as follows (lines 290-299):

*“During an acute P.vivax or P.falciparum infection, loss of RBCs occurs from haemolysis of both parasitized and unparasitized erythrocytes [10, 25-27]. The ratio of infected to uninfected RBCs lost is estimated at 1:34 for P.vivax and 1:8 for P. falciparum [28]. While the underlying mechanisms for the loss in unparasitized RBCs is not fully understood [29], it has been attributed to oxidative membrane damage induced by the malaria parasite [30]. G6PD plays a crucial role in maintaining RBC redox potential and reducing oxidative stress, thus as G6PD activity decreases with RBC age, older cells are preferentially removed from the peripheral blood [8]. Furthermore, parasite induced hemolysis results in a decrease in Hb concentration and oxygen delivery that triggers reactive erythropoiesis and production of young RBCs with higher G6PD activity [10], hence the mean age of the hosts RBC population decreases and the overall G6PD activity increases.”*

**6.	None of the studies included deficient cases in the acute phase of malaria which was when the study participants were recruited. So any analysis based on these classifications is inherently biased. Changes in G6PD activity per site per species across all study participants and the statistical significance should be shown. And possibly pooled for Ethiopia and Indonesia.**

This is not correct. One participant with deficient activities and 9 participants with intermediate activities were enrolled with acute malaria and revisited again after haematological recovery (Table 3).

G6PD deficient individuals were not excluded, and in fact individuals with lower activities were specifically targeted in Bangladesh. Our findings are consistent with aparasitaemic individuals with the lowest enzyme activity having the greatest increase in activity during acute infection – hence it is not surprising that there were so few individuals with G6PD intermediate or severe deficiency at enrolment. Indeed, these findings support previous studies highlighting that the proportion of patients with malaria having intermediate G6PD deficiency is significantly lower than that expected from cross sectional surveys of healthy population [6].

To clarify we have revised Table 3 to reflect the change in G6PD category between enrolment and follow up stratified by country. The only significant change in proportions of deficient, intermediate, and normal participants between enrolment and follow up is observed in Bangladesh. As stated above we hypothesise that this is due to the low proportion of deficient participants enrolled at the Ethiopian and Indonesian sites.

We have revised the discussion on lines 282-286 as follows:

*“There was a significant difference in participants G6PD status between enrolment and follow up, however this only reached statistical significance in Bangladesh where 45% of participants were G6PD deficient during follow up. In contrast only 1% of participants in Ethiopia and 7% in Indonesia had enzyme activities <70% during follow up.”*

**References**

1.	Ley B, Kibria MG, Khan WA, Auburn S, Phru CS, Jahan N, et al. Wide range of G6PD activities found among ethnic groups of the Chittagong Hill Tracts, Bangladesh. PLoS neglected tropical diseases. 2020;14(9):e0008697.

2.	Ley B, Kibria MG, Khan WA, Auburn S, Phru CS, Jahan N, et al. Wide range of G6PD activities found among ethnic groups of the Chittagong Hill Tracts, Bangladesh. PLoS Negl Trop Dis. 2020;14(9):e0008697. Epub 2020/09/15. doi: 10.1371/journal.pntd.0008697. PubMed PMID: 32925910; PubMed Central PMCID: PMCPMC7514097.

3.	Pfeffer DA, Ley B, Howes RE, Adu P, Alam MS, Bansil P, et al. Quantification of glucose-6-phosphate dehydrogenase activity by spectrophotometry: A systematic review and meta-analysis. PLoS Med. 2020;17(5):e1003084. Epub 2020/05/15. doi: 10.1371/journal.pmed.1003084. PubMed PMID: 32407380; PubMed Central PMCID: PMCPMC7224463 following competing interests: LvS receives a stipend as a Specialty Consulting Editor for PLOS Medicine and serves on the journal's editorial board.

4.	Price RN, Simpson JA, Nosten F, Luxemburger C, Hkirjaroen L, ter Kuile F, et al. Factors contributing to anemia after uncomplicated falciparum malaria. Am J Trop Med Hyg. 2001;65(5):614-22. PubMed PMID: 11716124.

5.	Douglas NM, Anstey NM, Buffet PA, Poespoprodjo JR, Yeo TW, White NJ, et al. The anaemia of Plasmodium vivax malaria. Malaria journal. 2012;11:135. doi: 10.1186/1475-2875-11-135. PubMed PMID: 22540175; PubMed Central PMCID: PMCPMC3438072.

6.	Ley B, Alam MS, Kibria MG, Marfurt J, Phru CS, Ami JQ, et al. Glucose-6-phosphate dehydrogenase activity in individuals with and without malaria: Analysis of clinical trial, cross-sectional and case-control data from Bangladesh. PLoS Med. 2021;18(4):e1003576. Epub 2021/04/24. doi: 10.1371/journal.pmed.1003576. PubMed PMID: 33891581; PubMed Central PMCID: PMCPMC8064587.
